# Rational engineering of xylanase hyper-producing system in *Trichoderma reesei* for efficient biomass degradation

**DOI:** 10.1186/s13068-021-01943-9

**Published:** 2021-04-08

**Authors:** Su Yan, Yan Xu, Xiao-Wei Yu

**Affiliations:** Key Laboratory of Industrial Biotechnology, Ministry of Education, School of Biotechnology, Jiangnan University, Wuxi, 214122 People’s Republic of China

**Keywords:** ACE1, Lignocellulose, *Trichoderma reesei*, Xylanase, Xylanolytic enzymes, XYNII, XYR1, Saccharification

## Abstract

**Background:**

Filamentous fungus *Trichoderma reesei* has been widely used as a workhorse for cellulase and xylanase productions. Xylanase has been reported as the crucial accessory enzyme in the degradation of lignocellulose for higher accessibility of cellulase. In addition, the efficient hydrolysis of xylan needs the co-work of multiple xylanolytic enzymes, which rise an increasing demand for the high yield of xylanase for efficient biomass degradation.

**Results:**

In this study, a xylanase hyper-producing system in *T. reesei* was established by tailoring two transcription factors, XYR1 and ACE1, and homologous overexpression of the major endo-xylanase XYNII. The expressed xylanase cocktail contained 5256 U/mL xylanase activity and 9.25 U/mL β-xylosidase (*p*NPXase) activity. Meanwhile, the transcription level of the xylanolytic genes in the strain with XYR1 overexpressed was upregulated, which was well correlated with the amount of XYR1-binding sites. In addition, the higher expression of associated xylanolytic enzymes would result in more efficient xylan hydrolysis. Besides, 2310–3085 U/mL of xylanase activities were achieved using soluble carbon source, which was more efficient and economical than the traditional strategy of xylan induction. Unexpectedly, deletion of *ace1* in C30OExyr1 did not give any improvement, which might be the result of the disturbed function of the complex formed between ACE1 and XYR1. The enzymatic hydrolysis of alkali pretreated corn stover using the crude xylanase cocktails as accessory enzymes resulted in a 36.64% increase in saccharification efficiency with the ratio of xylanase activity vs FPase activity at 500, compared to that using cellulase alone.

**Conclusions:**

An efficient and economical xylanase hyper-producing platform was developed in *T. reesei* RUT-C30. The novel platform with outstanding ability for crude xylanase cocktail production would greatly fit in biomass degradation and give a new perspective of further engineering in *T. reesei* for industrial purposes.

**Supplementary Information:**

The online version contains supplementary material available at 10.1186/s13068-021-01943-9.

## Background

Renewable resource lignocellulose consists of cellulose (40–60%) and hemicellulose (20–40%) [1], which shows significant importance for bioresource conversion and sustainable development. The biodegradation of lignocellulose for biofuel production needs the enzymatical hydrolysis that was mainly functioned by cellulase and hemicellulase. Several studies have been conducted to improve the hydrolysis efficiency by higher cellulase load and genetic manipulation of the cellulase producing strains for higher cellulase output [2, 3]. However, after pretreatment, resident hemicellulose (mostly xylan) could form a sheath on each cellulose microfibril in lignocellulose [4, 5] which makes a barrier for further hydrolysis [6]. Xylan is mainly composed of d-xylose, which is linked with β-1,4-glycosidic bonds, and other d-galactose and L-arabinose substituent groups, making a complex hetero-polymeric structure [1]. The biodegradation of xylan requires a complex enzyme cocktail that is commonly named xylanase [7]. Xylanase is a group of xylanolytic enzymes consisting of endo-xylanase, β-xylosidase, α-L-arabinofuranosidase, acetyl xylan esterase, and ferulic acid esterase, which decompose xylan into simple monosaccharide and xylooligosaccharides [1, 8], showing great potential in the feed, biorefinery, and pulp paper industry. Generally, the xylanase production is achieved by overexpression of endo-xylanases [9], which shows high catalyzing performance to the backbone of xylan. However, the unthorough de-branch of the side-chain group also hinders the cleavage efficiency of endo-xylanase [1, 10], which then influences the accessibility of cellulose, resulting in lower fermentable sugar yield for downstream biofuel production. Thus, it is wise to use xylanase cocktail for efficient biomass degradation, compared to addition of single kind of xylanase.

Filamentous fungus *Trichoderma reesei* was widely used as a workhorse for cellulase and xylanase fermentation. It is reported that 80.6 g/L extracellular protein, mainly composed of cellulase and xylanase, was produced in *T. reesei* through rational engineering [11]. Moreover, the genomic and transcriptional data from *T. reesei* showed the expression of multiple xylanolytic enzymes that belonged to different families and exhibited different cleave specificity to xylan [12–15], giving the potential to use *T. reesei* as a host for xylanase production in industrial scale. The main xylanases secreted under induction conditions are endo-xylanase XYNI and XYNII, which belong to the GH11 family and account for 90% of the secreted xylanase. The GH11 endo-xylanases act on xylan backbone and randomly cleave between β-1,4-glycosidic bonds, which termed as true xylanase. Among them, XYNII showed higher catalytic activity and stability [16, 17], giving better performance in industrial applications. Traditionally, the production of xylanases in *T. reesei* was achieved using the xylan-based substrate as the carbon source. However, the high cost and insolubility of xylan make unsatisfied and non-economical xylanase production, hindering further industrial fermentation.

The induction and expression of xylanolytic genes in *T. reesei* were co-regulated through multiple transcription factors (TFs). The widely investigated TF was the negative regulator CRE1, which controlled carbon catabolite repression (CCR) and could abolish the induction of cellulase and xylanase with the existence of easy-utilized carbon sources such as glucose [18]. Moreover, the transcription activator XYR1 served as a global TF that tightly controls the induction of cellulase and xylanase through specific binding to a GGC(A/T)_3_ motif in the promoter [19]. ACE1 serves as a negative factor for cellulase and xylanase production, deletion of *ace1* leads to increased production of cellulase and xylanase [20]. Besides, XPP1 [21] and SxlR [22] were also shown as the negative TFs for a few xylanases (XYN1, XYN2, XYN5, BXL2), deletion of which would cause an increase in xylanase production.

The recombinant expression of xylanase in *T. reesei* has already been performed using different promoters including *Pxyn2*, *Pegl1*, *Pcbh2*, *Pcbh1*, and *Ppdc*, and achieved different results [23–25]. The strongest cellobiohydrolase I promoter *Pcbh1* that was highly induced with cellulosic carbon source has been widely used for recombinant expression in *T. reesei* [26, 27], while de Faria et al*.* made a low recombinant xylanase production using *Pcbh1* which might due to the low catalytic activity of xylanase and the degradation of foreign protein [24]. Li et al. made the highest xylanase activity of 9266 U/mL through the homologous expression of native XYNII under the strong pyruvate decarboxylase (*pdc*) promoter with a high concentration of glucose (7%) [28]. However, the strategy of the recombinant expression of XYNII in *T. reesei* QM9414 (a carbon catabolite repressed strain) only increased the endo-xylanase activity, further application in xylan degradation and other application needs the addition of other associated xylanolytic enzymes. Therefore, it is requisite to build a xylanase hyper-producing system that could produce different kinds of xylanases for efficient xylan and biomass degradation.

In this study, we developed a strategy that combined not only regulation of two transcription factors, XYR1 and ACE1, to obtain higher expression of xylanolytic enzymes, but also the homologous expression of the major xylanase XYNII under the P*cbh1* promoter in *T. reesei* for improved production of endo-xylanase. The xylanase cocktail produced by the recombinant strain was rich in endo-xylanase, associated with enhanced xylanolytic enzymes to limit the side-chain inhibition of xylan. Besides, the recombinant *T. reesei* could also achieve higher xylanase production in other economical carbon sources such as lactose or glucose medium, suggesting a more economical-friendly and flexible strategy for xylanase production. Moreover, the optimal ratio of xylanase *vs* cellulase for saccharification was also analyzed for higher saccharification efficiency. Our study would give a new perspective for efficient biomass degradation and meet the increased demand for biofuel production.

## Results

### Effects of XYR1 overexpression and *ace1* disruption on xylanase production

**Fig. 1:**
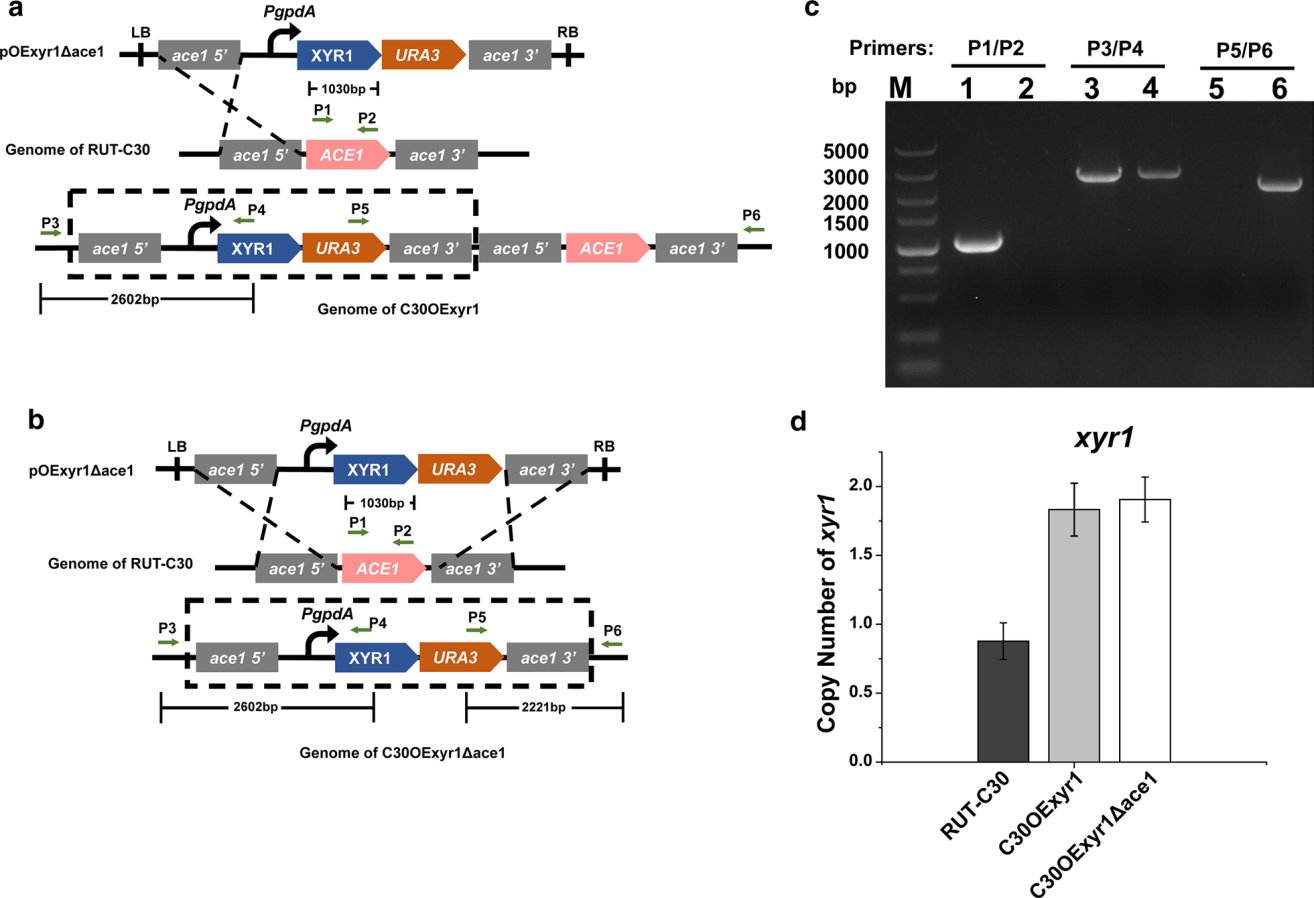
The scheme for XYR1 overexpression and ACE1 disruption*. a* A scheme for C30OExyr1 construction. **b** A scheme for C30OExyr1Δace1 construction. **c** PCR analysis of recombinant C30OExyr1 and C30OExyr1Δace1. Lanes 1, 3, and 5 represent the PCR results of C30OExyr1, and Lanes 2, 4, and 6 represent the PCR results of C30OExyr1Δace1, which were detected using three pair primers indicated by arrows. **d** The copy number of *xyr1* which was assayed by qPCR using the genomic DNA of RUT-C30, C30OExyr1 and C30OExyr1Δace1 as template, the gene *sar1* was represented as single copy

In *T. reesei*, the induction of xylanase was triggered by cellulose, xylan and its polysaccharides. Meanwhile, the expression of xylanolytic genes was also regulated by transcription factors. To enhance the expression of different xylanolytic genes and increase xylanase production, we choose two well-characterized transcription factors, XYR1 and ACE1, which act as an activator and repressor for cellulase and xylanase production, respectively. As intended, we perform a combined strategy that upregulates activator XYR1 and downregulates the repressor ACE1 through inserting XYR1 overexpression cassette into *ace1* locus. Transformant with correct insertion into *ace1* locus was verified by PCR (Fig. 1b, c, lanes 2, 4, and 6) and termed as C30OExyr1Δace1. Simultaneously, a transformant harbored XYR1 overexpression cassette in *ace1* locus by single-crossover homologous recombination was also constructed and termed as C30OExyr1, leaving an intact *ace1* in the chromosome (Fig. 1a, c, lanes 1, 3, and 5). The scheme for the transformants construct was indicated in Fig. 1a, b. The transformants both showed a similar growth trend compared to the parent strain on the PDA plate (data not shown). In addition, the copy number of *xyr1* cassette in the parent strain and two transformants were also verified by qPCR (Fig. 1d) with primer listed in Additional file 1: Table S3.

**Fig. 2:**
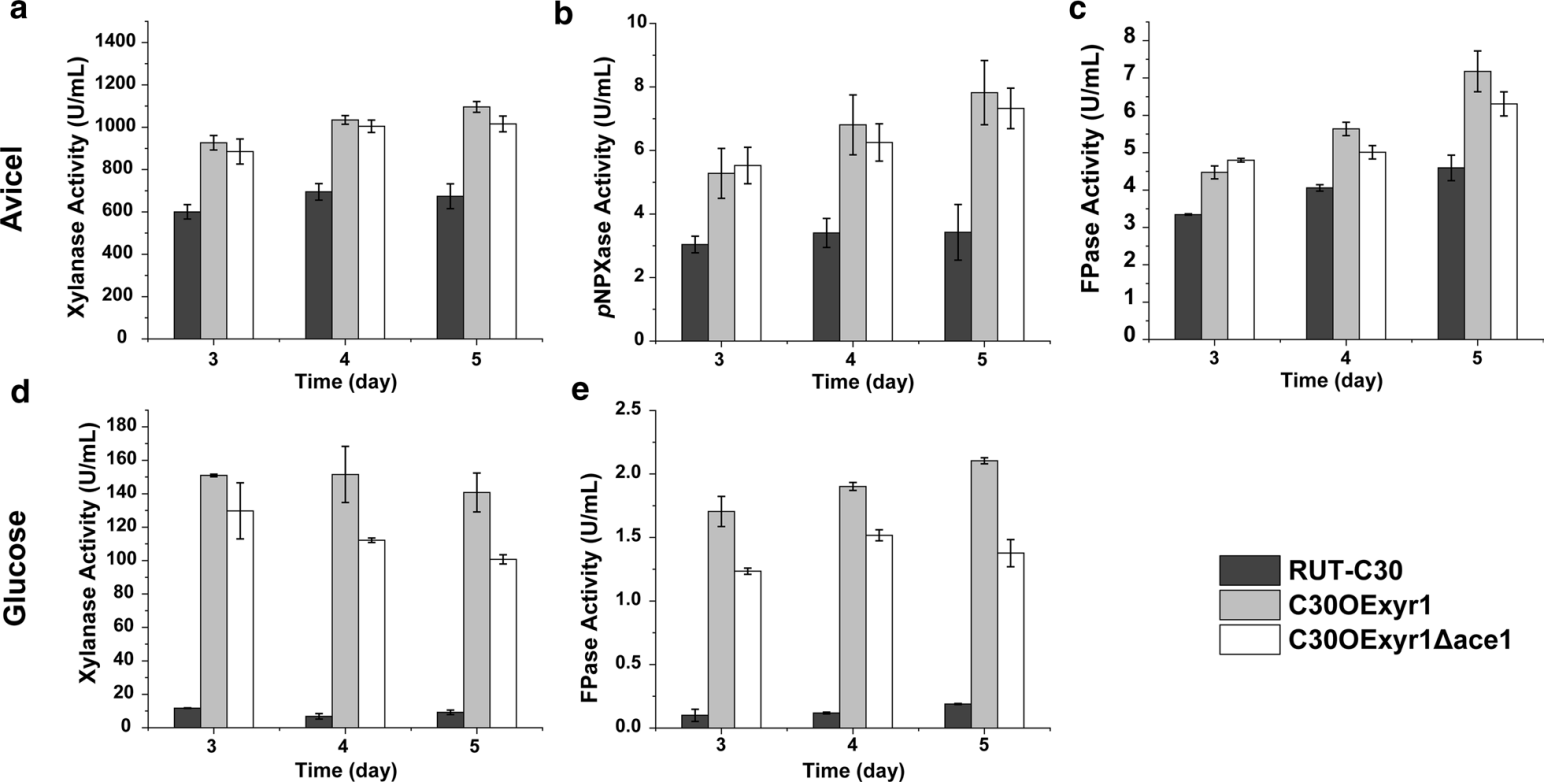
Effects of XYR1 overexpression and ACE1 disruption on the FPase, xylanase and *p*NPXase activities. **a–c** The xylanase *p*NPXase and FPase activities of the recombinant strains RUT-C30, C30OExyr1 and C30OExyr1Δace1 cultured with 3% Avicel. **d, e** The xylanase and FPase activities of RUT-C30, C30OExyr1 and C30OExyr1Δace1 in 5% glucose medium. For each experiment, three individual replicates were performed

Then, the xylanase-producing performance of the transformants and parent strain was analyzed through culturing three strains in Avicel-inducing medium. The result shows that the xylanase activity in C30OExyr1 and C30OExyr1Δace1 is significantly increased compared to that of RUT-C30 (Fig. 2a). In addition, the highest xylanase activity of 1095 U/mL was achieved in C30OExyr1 on day 5, which was 1.62-fold higher than that of RUT-C30 (Fig. 2a). Meanwhile, for further investigation of the xylanolytic enzymes, the β-xylosidase (*p*NPXase) activity was also analyzed. As shown in Fig. 2b, the *p*NPXase activity in C30OExyr1 achieved 7.82 U/mL on day 5, which was 2.29-fold higher than that in RUT-C30. However, beyond expectation, the xylanase activity in C30OExyr1Δace1 (1015 U/mL) on day 5 is a little bit lower than that of C30OExyr1 (1095 U/mL) (Fig. 2a). In addition, similar results were also attained for *p*NPXase activity (Fig. 2b). Although the little discrepancy in our result did not show statistical significance, the biological repetition in different batch cultures was also conducted, which shows similar trends to the results in Fig. 2a, b, indicating that the ACE1 disruption in strain C30OExyr1 hardly give a positive effect as reported by Rauscher et al*.* [29]. Moreover, to test whether the unexpected result specifically affects xylanase and xylanolytic enzymes, we also test the cellulase activity as indicated by FPase activity. As shown in Fig. 2c, a similar result shows the ACE1 disruption in strain C30OExyr1 would give lower cellulase and xylanase output, rather than further enhance the production. Besides, to exclude the negative effect caused by ACE1 disruption in our study, C30Δace1 was also constructed and analyzed for cellulase and xylanase activity. Similar to the results achieved by Aro et al*.* [20], the cellulase and xylanase activity in C30Δace1 was higher than that in RUT-C30 (data not shown). The results show that the negative effect of ACE1 deletion is only present when XYR1 is overexpressed.

Furthermore, to test whether the decreased xylanase activity is carbon source dependent, the FPase and xylanase activities of C30OExyr1Δace1, C30OExyr1, and their parent strain RUT-C30 were analyzed in a repression medium that glucose was used as the sole carbon source. As expected, the cellulase and xylanase activity in RUT-C30 is almost repressed due to the CCR as reported previously [18] (Fig. 2d, e). While for C30OExyr1, the xylanase activity of 151.5 U/mL was achieved on day 4 due to the release of CCR, which was 13.7-fold higher than that of RUT-C30 (Fig. 2d). In addition, the cellulase and xylanase activity of C30OExyr1 was higher than that of C30OExyr1Δace1 (Fig. 2d, e), which was similar to the trend in Avicel medium, suggesting that the negative effect of *ace1* disruption while XYR1 overexpression is carbon source independent.

### Transcription profiles of xylanolytic genes in C30OExyr1 and C30OExyr1Δace1

To give a deep understanding of the decreased xylanase activity by ACE1 disruption with XYR1 overexpression, and to estimate the expression of other xylanolytic genes which contributed to better xylan utilization in two transformants C30OExyr1Δace1 and C30OExyr1, seven xylanolytic genes were selected for further study. The analyzed xylanolytic genes include *xyn1* (jgi|Trire2:74223), *xyn2* (jgi|Trire2:123818) that encode for two major GH11 endo-xylanases, which were highly active on unbranched xylan backbone; *xyn3* (jgi|Trire2:120229) that encodes for a GH10 endo-xylanase, prefer to hydrolyze short and branched xylan; *xyn4* (jgi|Trire2:111849) encoding for GH30 xylanase harbored both exo- and endo-xylanase activity; *bxl1* (jgi|Trire2:121127) encoding for β-xylosidase, act on xylooligosaccharides and release xylose residue; *axe1* (jgi|Trire2:73632) and *abf1* (jgi|Trire2:123283) that encode for acetyl xylan esterase and α-L-arabinofuranosidase, respectively, which degrade side chain of xylan.

**Fig. 3:**
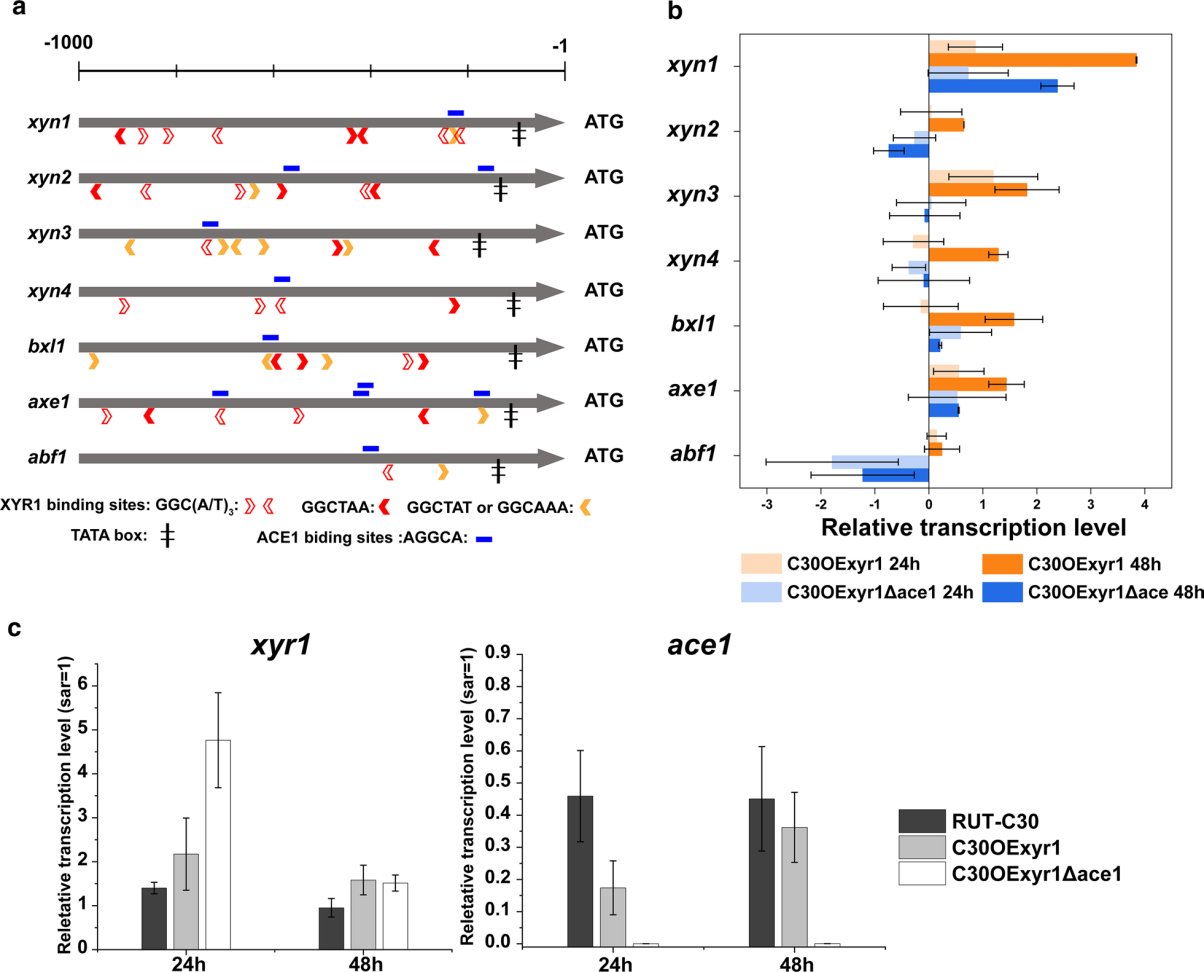
Transcription analysis of C30OExyr1 and C30OExyr1Δace1.** a** In silico analysis of the putative binding sites of XYR1, ACE1 in the 1000 bp upstream of xylanolytic genes. The promoter sequence was acquired from the *T. reesei* genomic database. https://genome.jgi.doe.gov/Trire2/Trire2.home.html. The binding sites of XYR1 were searched with GGC(A/T)_3_ motif, the strongest binding of GGCTAA and stronger binding of GGCAAA and GGCTAT were additionally indicated. The putative ACE1 binding sites were searched by the AGGCA core sequence. **b** The relative transcription level of xylanolytic genes. The transcription level of the corresponding gene of RUT-C30 was set as 1, and the transcription level was indicated by log_2_(fold change). **c** The relative transcription level of *xyr1* and *ace1.* The transcription level was measured using the 2^−ΔΔCT^ method, and the transcription level of *sar1* was used for normalization. All the strains were cultured with 3% Avicel, samples at 24 h and 48 h were analyzed for RT-qPCR, and the experiments were conducted with three biological repetitions

For transcription analysis, two transformants C30OExyr1Δace1, C30OExyr1 and their parent strain RUT-C30 were cultured in Avicel-inducing medium, and mycelia were sampled at 24 h and 48 h for analysis. First, the transcription level of *xyr1* was measured and the elevated transcription level of *xyr1* in C30OExyr1Δace1 and C30OExyr1 means the constitutively expressed XYR1 (Fig. 3c). Significantly, the transcription of *xyr1* of C30OExyr1Δace1 at 24 h was 2.19-fold higher than that in C30OExyr1 (Fig. 3c). Mach-Aigner et al*.* [30] demonstrated that the transcription of *xyr1* was repressed by ACE1, which was highly similar to our result, indicating that higher transcription of *xyr1* in this ACE1 disrupted transformant C30OExyr1Δace1 might be attributed to the de-repression of ACE1. Meanwhile, the transcription level of *ace1* was also determined, the result in Fig. 3c shows a comparable transcription of *ace1* in strain C30OExyr1 and RUT-C30 at 48 h. The undetectable transcription of *ace1* in C30OExyr1Δace1 further confirmed the complete loss of ACE1 function.

The analysis of the transcription level of seven xylanolytic genes demonstrated that the transcription of selected genes was highly upregulated in strain C30OExyr1 compared to that in C30OExyr1Δace1 (Fig. 3b). Although higher transcription of *xyr1* could result in higher transcription of downstream genes [31], the higher transcription level of *xyr1* in C30OExyr1Δace1 no more give a higher transcription of downstream xylanolytic genes (Fig. 3b, c), suggesting that the loss of ACE1 function in C30OExyr1 might influence the regulation of XYR1.

For a detailed analysis of the enhanced transcription of xylanolytic genes, in silico analysis was also combined. We first explored the 1000 bp upstream region of seven xylanolytic genes for the GGC(A/T)_3_ motif which represents the binding sites of regulator XYR1 [32], the motif before the TATA box is considered as the putative regulator binding sites of XYR1. As indicated in Fig. 3a, we identified nine XYR1-binding sites (XBD) in the 1000 bp upstream of *xyn1*. Among nine XBDs, three XBDs were reported as the best binding motif of XYR1 with the sequence of GGCTAA [32], and the preferable XBD GGCAAA was also detected in the promoter of *xyn1*, which was well correlated with the 3.84-fold increase in the transcription level of *xyn1* at 48 h (Fig. 3b). Besides, the transcription level of *xyn3*, *xyn4*, *bxl1*, and *axe1* was upregulated at least 1.28- to 1.81-fold in C30OExyr1 at 48 h. In silico analysis revealed that from four to eight XBDs with a varied amount of GGCTAA/GGCTAT/GGCAAA motif was lied in the upstream region of these xylanolytic genes, indicating the strong transcription activation in strain C30OExyr1. Moreover, the lower transcription activation of *abf1* in C30OExyr1 was consistent with fewer XBDs in its upstream region, and no GGCTAA motif was presented in this region, suggesting that *abf1* was only weakly regulated by XYR1. However, the transcription of *xyn2* which encodes for the major endo-xylanase was only increased by 0.56-fold at 48 h, the less pronounced increase in *xyn2* transcription was not well correlated with seven XBDs with three GCCTAA motif in the upstream region (Fig. 3a, b). For strain C30OExyr1Δace1 that lacks intact *ace1*, the upregulated transcription level of *xyn1*, *bxl1* and *axe1* were detected both at 24 h and 48 h, which was consistent with the enzyme activity data (Fig. 2a, b). While the transcription level of *xyn2*, *xyn3*, *xyn4* and *abf1* was unchanged or downregulated compared to RUT-C30. Although several ACE1 binding sites were also detected in these xylanolytic genes, the transcription data did not show any correlation between transcription strength and the number of binding sites.

### Further enhancing xylanase production by XYNII overexpression

**Fig. 4:**
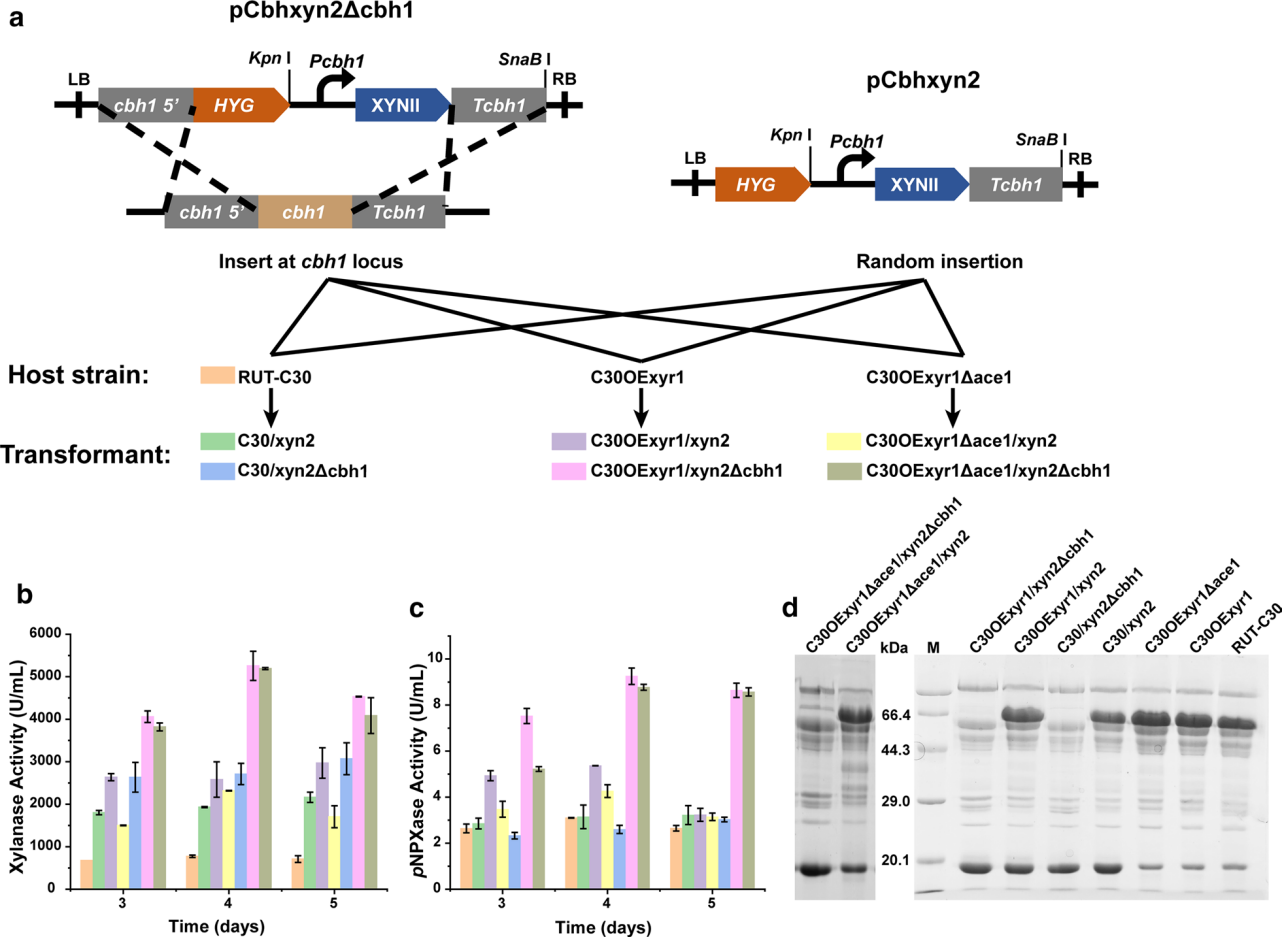
The xylanase profile of recombinant strains with XYNII overexpression.** a** The scheme of XYNII overexpression in RUT-C30, C30OExyr1 and C30OExyr1Δace1. **b, c** The xylanase and *p*NPXase activities of the recombinant strains cultured with 3% Avicel. **d** The SDS-PAGE analysis of different recombinant strains cultured with 3% Avicel. 3.75 μL supernatant of transformants was loaded for each lane. The band near 20.1 kDa was the corresponding band of XYNII. For each experiment, three individual replicates were performed

In strain C30OExyr1, the transcription level of *xyn1*, *xyn3*, *xyn4*, *bxl1*, and *axe1* were upregulated which lead to enhanced xylanase production in Avicel induction condition, however, the transcription level of major endo-xylanase *xyn2* was only increased 0.56-fold (Fig. 3b). In general, the GH11 endo-xylanase was termed as true xylanase due to their high catalytic ability towards xylan backbone [10], suggesting its significant role in xylan degradation. Among that, the specific activity of XYNII was 16-fold than that of XYNI [16], showing potential for xylan degradation. To further increase the capacity to cleave xylan backbone, we use the cellulose-induced promoter *Pcbh1* [26, 27], which was the strongest promoter in *T. reesei*, to increase the production of endo-xylanase. The XYNII expression cassette was constructed as described in “Methods”, and the cassette was transformed into C30OExyr1, C30OExyr1Δace1 and RUT-C30 to compare the xylanase-producing ability in the different strains. Meanwhile, for higher production, we also use a cassette that harbors *cbh1* homologous arms for site-directed insertion in the *cbh1* locus, resulting in 6 recombinants for further analysis (Fig. 4a).

**Fig. 5:**
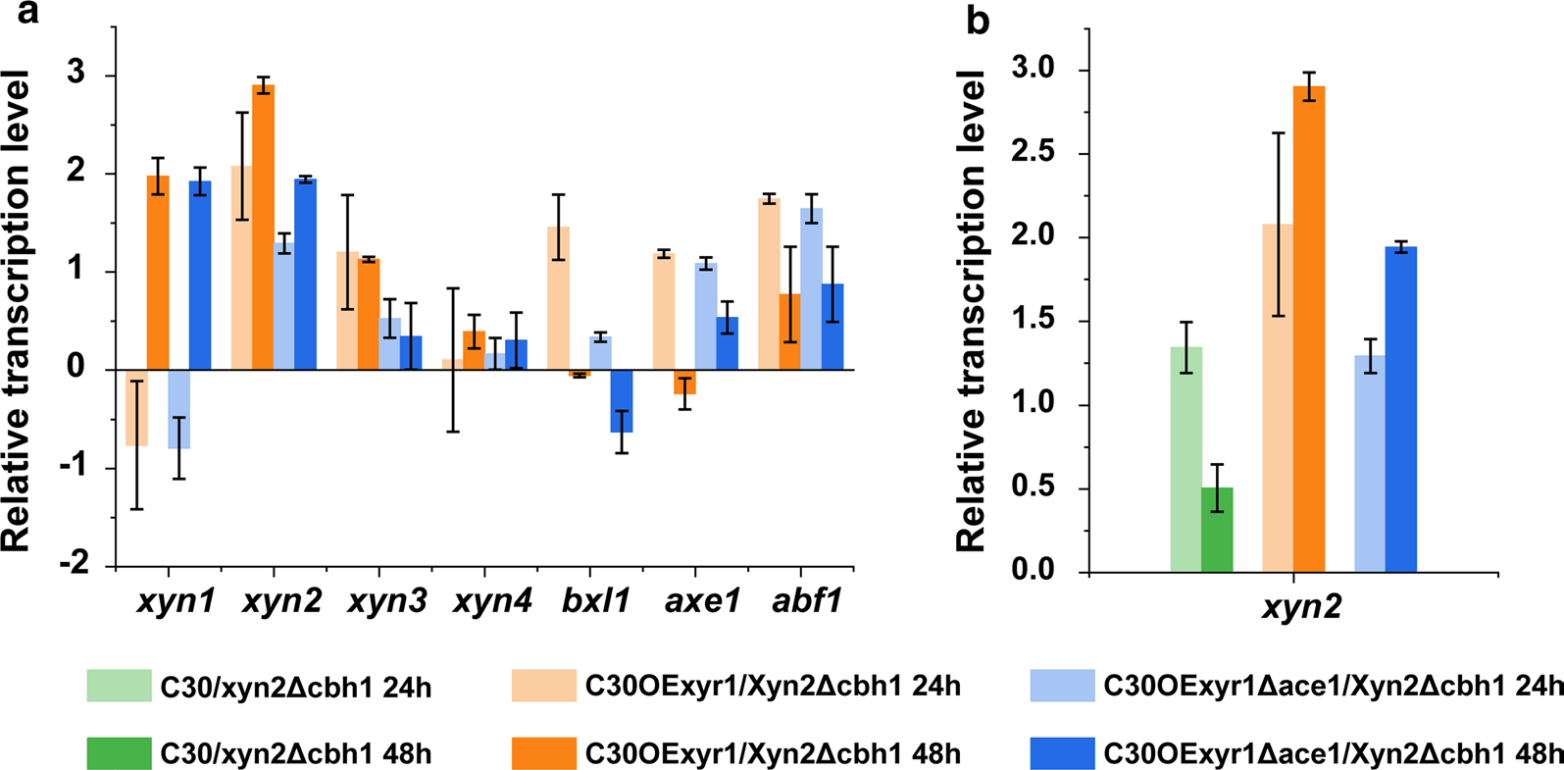
Transcription level of xylanolytic genes in C30/xyn2Δcbh1, C30OExyr1/xyn2Δcbh1 and OExyr1Δace1/xyn2Δcbh1. **a** The relative transcription level of xylanolytic genes in C30OExyr1/xyn2Δcbh1 and C30OExyr1Δace1/xyn2Δcbh1 cultured on Avicel. **b** The relative transcription level of *xyn2* in C30/xyn2Δcbh1, C30OExyr1/xyn2Δcbh1 and C30OExyr1Δace1/xyn2Δcbh1. The strains were all cultured in the Avicel medium and sampled at 24 h and 48 h, respectively. The transcription level of the corresponding gene of RUT-C30 was set as 1, and the transcription level was indicated by log_2_(fold change). All the experiments were conducted with three biological replicates

Xylanase production of different strains was performed by induction with 3% Avicel. The result shows that the xylanase activity in all transformants was significantly higher than that of RUT-C30. As expected, the transformants with a cassette inserted into *cbh1* locus by homologous recombinant could produce higher xylanase activity than the transformants with random insertion (Fig. 4b), which was consistent with the previous study [33]. The higher XYNII output and the absence of CBHI were also verified by SDS-PAGE (Fig. 4d). The transformant C30/xyn2Δcbh1, that the XYNII expression cassette was inserted into *cbh1* locus in RUT-C30, achieved 3069 U/mL xylanase activity after 5 day’s induction, which was 4.33-fold higher than that in RUT-C30, revealing a dramatic increase in xylanase activity due to the overexpression of XYNII. The highest xylanase activity of 5256 U/mL was achieved by C30OExyr1/xyn2Δcbh1 at day 4, which was 6.79- and 1.71-fold higher compared to that of RUT-C30 and C30/xyn2Δcbh1, respectively (Fig. 4b). Similar to the result presented in Fig. 2, the transformant C30OExyr1Δace1/xyn2Δcbh1 produced much less xylanase activity than C30OExyr1/xyn2Δcbh1 (Fig. 4b). Moreover, the transcription level of *xyn2* was also measured in three strains. Results show that the transcription level of *xyn2* in C30OExyr1/xyn2Δcbh1 was higher than that in C30OExyr1Δace1/xyn2Δcbh1 and C30/xyn2Δcbh1 either at 24 h or 48 h (Fig. 5b), which was consistent with the xylanase activity.

For analyzing other xylanolytic enzymes, the *p*NPXase activity was also detected. As indicated in Fig. 4c, comparative *p*NPXase activity was achieved in strains C30/xyn2, C30/xyn2Δcbh1 and RUT-C30. The *p*NPXase activity in other transformants was much higher than that in RUT-C30 due to the overexpression of XYR1. In addition, 9.25 U/mL *p*NPXase activity was achieved in C30OExyr1/xyn2Δcbh1, which was about 2.98-fold higher than that of RUT-C30 (Fig. 4c). Not only that, the *p*NPXase activity achieved in C30OExyr1/xyn2Δcbh1 (9.25 U/mL) was higher than that in C30OExyr1 (7.82 U/mL) (Figs. 2b, 4c), suggesting that the transformant C30OExyr1/xyn2Δcbh1 could produce more abundant xylanolytic enzymes that were preferable for xylan degradation. Meanwhile, the transcription analysis of xylanolytic gene in C30OExyr1/xyn2Δcbh1 and C30OExyr1Δace1/xyn2Δcbh1 also shows the upregulation of seven selected xylanolytic genes (Fig. 5a), especially in *xyn1* and *xyn2*, which means a significant increase in GH11 endo-xylanase activity. Meanwhile, the transcription level of *xyn3*, *bxl1*, *axe1* and *abf1* was also increased by at least onefold in C30OExyr1/xyn2Δcbh1 (Fig. 5a), suggesting the upregulation of different xylanolytic genes. However, the transcription level of *xyn4* was poorly activated in C30OExyr1/xyn2Δcbh1 (Fig. 5a), which was inconsistent with the result in C30OExyr1 (Fig. 3b). Similar to the data in Fig. 3b, the transcription of xylanolytic genes above was weakly upregulated in C30OExyr1Δace1/xyn2Δcbh1 than C30OExyr1/xyn2Δcbh1, resulting in less pronounced xylanase-producing ability.

In summary, we use the strong cellulose-induced promoter *Pcbh1* to increase the endo-xylanase activity of *T. reesei*. Compared to other strains constructed above, the transformant C30OExyr1/xyn2Δcbh1 with XYNII expression at *cbh1* locus gives the highest xylanase-producing ability with strong expression of xylanolytic enzymes, showing potential for efficient biomass degradation. The xylanase production in different organisms was also compared (Additional file 1: Table S4), and the xylanase activity and β-xylosidase activity produced in our study were both prominent among other studies.

### High-level xylanase production was achieved using soluble carbon source in C30OExyr1/xyn2Δcbh1

Lactose and glucose were widely used as easily utilized, soluble and economical carbon sources for industrial fermentation. In *T. reesei*, lactose only moderately induces production of cellulase and xylanase [34] while glucose has been widely reported as a repressor due to CCR [18]. Then, in this section, the xylanase production was also assessed by culturing transformant C30OExyr1/xyn2Δcbh1 with lactose or glucose as sole carbon source, the xylanase activity and *p*NPXase activity were detected as described in “Methods”.

**Fig. 6:**
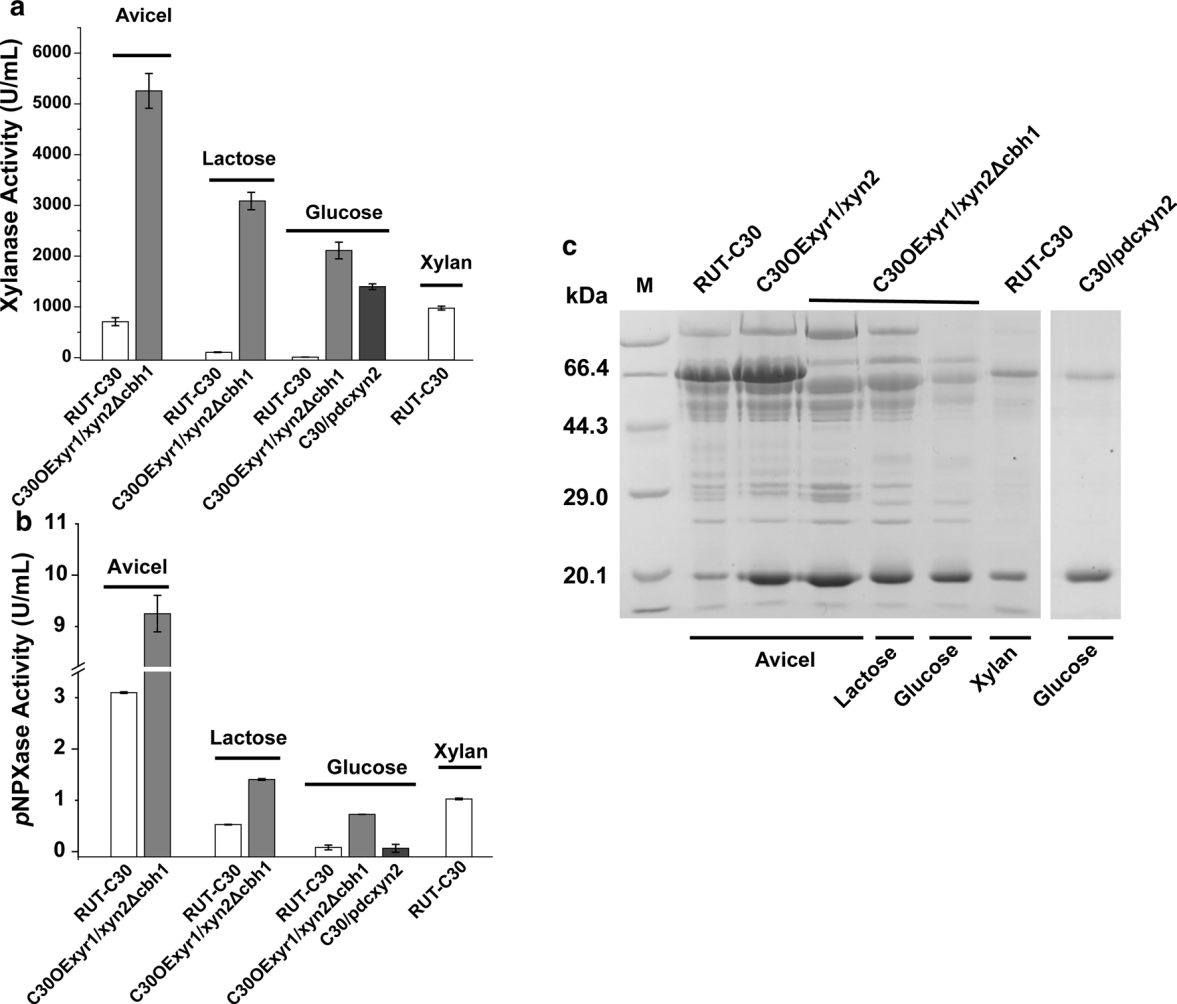
Xylanase production comparison between different strains cultured with different mediums. The xylanase production of C30OExyr1/xyn2Δcbh1 was conducted in lactose or glucose medium, as well. Meanwhile, the transformant C30/pdcxyn2 which was constructed using the strongest constitutive promoter *Ppdc* was also applied for xylanase production. Besides, the xylanase production using the traditional strategy of xylan induction was also conducted in RUT-C30. Strains were cultured with 3% Avicel, 5% lactose, 5% glucose or 3% xylan according to intention. The xylanase activity **(a)** *p*NPXase activity **(b)** and SDS-PAGE **(c)** analysis of supernatant were analyzed

When lactose was used as the sole carbon source, the highest xylanase activity reached 3085 U/mL in C30OExyr1/xyn2Δcbh1, and the *p*NPXase activity was also increased to 1.41 U/mL, which was 29.1-fold and 2.66-fold than that of RUT-C30 (Fig. 6a, b). When cultured with glucose as the carbon source, 2310 U/mL xylanase activity and 0.84 U/mL *p*NPXase activity was achieved in C30OExyr1/xyn2Δcbh1. While in parent strain RUT-C30, the xylanase production was mostly repressed, that 11 U/mL xylanase activity and 0.08 U/mL *p*NPXase activity was detected, indicating a dramatic increase of xylanase production caused by the release of CCR. As well, the transcription level of xylanolytic genes was also measured, which shows an increased expression in xylanolytic enzymes either in lactose or glucose medium (Additional file 1: Figure S1), revealing that the xylanolytic enzymes could also be highly induced using lactose or glucose as carbon source. Glucose is an important carbohydrate that could participate in cellular metabolism, and the higher concentration of glucose could provoke higher transcription of pyruvate decarboxylase (*pdc*) [28]. Li et al*.* achieved 9266 U/mL xylanase activity by constitutive expression of XYNII using this strong promoter *Ppdc.* To compare the xylanase production of C30OExyr1/xyn2Δcbh1 and the strain with constitutive expression of XYNII, we also constructed a strain using *Ppdc* promoter to initiate the transcription of *xyn2* and termed as C30/pdcxyn2*.* Unexpectedly, as indicated in Fig. 6a, the xylanase production of 1398 U/mL was achieved cultured with 5% glucose, which was much lower than that of C30OExyr1/xyn2Δcbh1 in the same condition. Meanwhile, the *p*NPXase activity of C30/pdcxyn2 was also analyzed, showing complete repression as same as RUT-C30 (Fig. 6b).

In general, the induction of xylanase was performed using xylan as the inducer in *T. reesei*. Then, the xylanase production under xylan medium was also determined in the parent RUT-C30 by culturing strains with 3% xylan. It was obvious that the xylanase production on xylan was higher than that on Avicel (Fig. 6a). However, the *p*NPXase activity under xylan was nearly one-third of that on Avicel, suggesting the expression of β-xylosidase was more preferable on the cellulosic substrate. Besides, the xylanase production in C30OExyr1/xyn2Δcbh1 under different carbon sources was much higher than that of RUT-C30 cultured on xylan, which shows the better capacity for xylanase production in C30OExyr1/xyn2Δcbh1. The SDS-PAGE analysis of xylanase production of different strains with different carbon was also conducted as indicated in Fig. 6c. The results show that the band near 20 kDa, referred to XYNII, was most abundant in C30OExyr1/xyn2Δcbh1 than other strains discussed above.

### Synergistic effect with the addition of xylanase cocktails of C30OExyr1/xyn2Δcbh1

The addition of xylanase into cellulase, that termed as synergistic interaction, could improve the saccharification efficiency of biomass [6], resulting in higher reducing sugar yield. However, the addition of xylanase into the cellulase system for better saccharification requires further addition of enzyme, which increases the enzyme load and cost for saccharification (Table 1) [35, 36]. To systematically explore the appropriate rate of xylanase and cellulase in saccharification, we calculated the total cellulase and xylanase activity in each reaction as described in method section, and use a criterion *X*/*F*, the ratio of xylanase activity *vs* FPase activity, to evaluate the amount of xylanase and cellulase in the saccharification system. The profile of enzyme preparation is listed in Table 2. The alkali pretreated corn stover (APCS) was used as substrate for saccharification and equal amount (protein concentration) of enzyme was loaded in each reaction, and a series of *X*/*F* from 24 to 2770 were set as indicated in Table 3.

**Table 1:** Improvement of lignocellulose degradation by addition of xylanase in the literature

Biomass^a^	Cellulase loaded without xylanase addition	With xylanase addition		Improvement^b^ (%)	Reference
		Cellulase loaded	Xylanase loaded		
APCS	8 mg/g biomass (12.2 FPU/g biomass)	5.48 mg/g biomass (8.4 FPU/g biomass)	2.53 mg/g biomass	34.64	This study
SPB	2.1 mg/g biomass	2 mg/g biomass	0.1 mg/g biomass	31.10	[49]
SPCS	35 mg/g cellulose	5 mg/g cellulose	30 mg/g cellulose	19.70	[6]
SPCS	5 mg/g cellulose	5 mg/g cellulose	30 mg/g cellulose	~ 41	
SPSB				~ 31	
SPLP				~ 15	
RTILs-PWS	50 mg/g biomass	50 mg/g biomass	50 IU/g biomass	56.71	[50]
APCS	25 FPU/g biomass	25 FPU/g biomass	200 U/g biomass	90.20^c^	[36]
APCS	1.38 FPU/g biomass	1.38 FPU/g biomass	1.06 mg/g biomass	55.60^d^	[51]
SPRS	1 mL cellulase (sigma)	1 mL cellulase (sigma)	0.42 mg/g wet biomass	12.40	[35]

^a^APCS: alkali pretreated corn stover; SPB: alkaline pretreated bagasse; SPCS: steam pretreated corn stover; SPSB: steam pretreated sweet sorghum bagasse; SPLP: steam pretreated lodgepole pine; RTILs-PWS: RTILs (1-ethyl-3-methylimidazolium acetate) pretreated wheat straw; SPRS: steam pretreated rice straw

^b^The improvement means the improvement in released glucose with xylanase addition compared to that without xylanase addition

^c^The improvement of 90.2% was measured after 8 h saccharification, which released 14 mM reducing sugar

^d^The improvement of 55.60% finally makes a 46.70% hydrolysis of cellulose

**Table 2:** The specific activity of enzyme preparation

	Protein concentration (mg/mL)	FPase (U/mL)	*p*NPGase (U/mL)	Xylanase (U/mL)	*p*NPXase (U/mL)
Cellulase^a^	2.56	3.93	3.72	93.93	0.78
Xylanase^b^	2.22	1.57	1.80	4298.24	7.37

^a^The commercial cellulase used in this study is purchased from Sunson (NingXia, China)

^b^The xylanase cocktail was produced by culturing C30OExyr1/xyn2Δcbh1 in Avicel medium for 5 days

**Table 3:** The detailed content of enzyme preparation

*X*/*F*^a^	Cellulase volume (mL)	Total FPase activity (U)	Xylanase volume (mL)	Total xylanase activity (U)	Improvement^b^ (%)
24	1.56	6.14	0	146.53	100.00
50	1.53	6.07	0.04	315.64	110.82 ± 2.19
100	1.47	5.94	0.11	610.88	114.99 ± 1.63
200	1.36	5.71	0.24	1159.32	126.14 ± 3.91
500	1.07	5.09	0.57	2550.50	134.63 ± 5.37
1000	0.70	4.32	0.99	4321.01	130.58 ± 3.31
1500	0.43	3.75	1.30	5628.10	120.90 ± 4.50
2000	0.23	3.31	1.54	6640.89	111.10 ± 3.27
2770	0	2.83	1.80	7735.83	85.48 ± 7.20

^a^*X*/*F* means the xylanase activity vs FPase activity in the saccharification system. And equal amount of enzyme (8 mg/g APCS) was loaded for each reaction

^b^Improvement refers to the improved glucose yield compared to that using cellulase alone (*X*/*F* = 24)

**Fig. 7:**
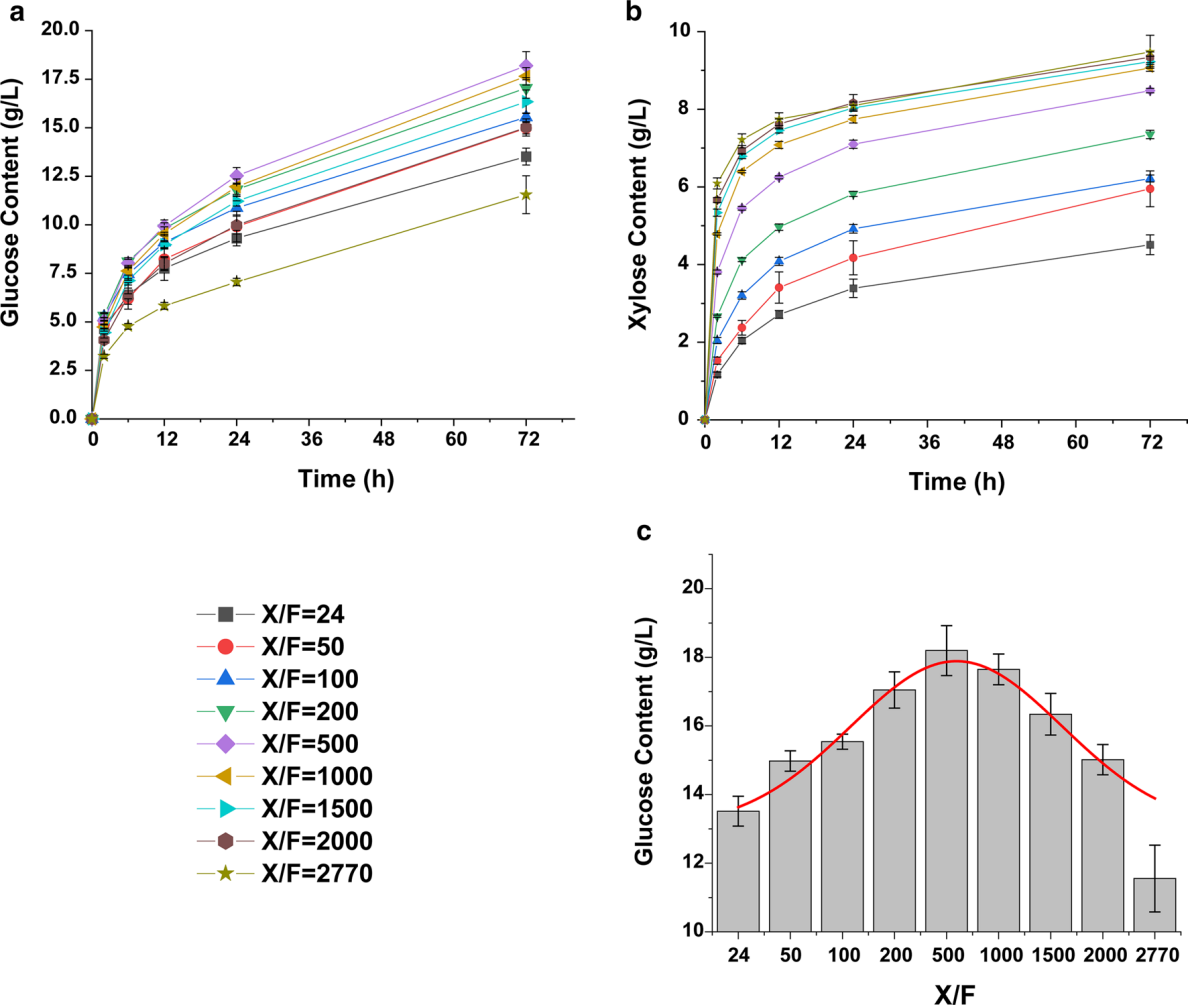
Saccharification of APCS with xylanase cocktail produced by C30OExyr1/xyn2Δcbh1 as the accessory enzyme. The glucose content **(a)** and xylose content **(b)** of different saccharification groups were analyzed by HPLC, an equal amount of enzyme load (8 mg/g APCS) was applied. The comparison of glucose content after 72 h hydrolysis was also presented **(c)**. Three individual biological repeats were performed for each reaction

As expected, the result shows that the xylose content was continuously increased with the increasing proportion of xylanase. The highest xylan hydrolysis efficiency of 91.08% was achieved at the *X*/*F* = 2770, giving the highest xylose yield of 9.48 mg/mL (Additional file 1: Table S5). With higher xylanase addition, the cellulase proportion and total cellulase activity were continuously decreased, while the glucose yield was significantly improved with appropriate xylanase addition (Fig. 7a, Table 3). In addition, the highest glucose yield (18.20 mg/mL), which was corresponding to the cellulose hydrolysis of 63.59%, was reached with *X*/*F* = 500, which was 34.64% higher than that using cellulase alone (Table 3, Additional file 1: Table S5). At *X*/*F* = 500, the cellulase addition was only 68.6% of that using cellulase alone, while the glucose yield gave a 34.64% increase, compared to that of *X*/*F* = 24. Although further increased xylanase proportion could also improve glucose yield than that using cellulase alone (*X*/*F* = 24), the glucose yield was continuously reduced with *X*/*F* beyond 500 (Fig. 7a, c). In addition, when *X*/*F* reached 2000, the cellulase load was only 1.18 mg/g biomass, which was 14.75% of that using cellulase alone, and the total FPase activity for saccharification was 3.31 U, which was about 54% of that at *X*/*F* = 24, while the glucose yield was still 1.11-fold higher than that using cellulase alone (Fig. 7c, Table 3). Moreover, further increase in xylanase proportion gave a total 15% decrease in glucose yield, which might be attributed to the unbalanced proportion of cellulase and xylanase.

## Discussion

The crucial step of lignocellulose utilization for renewable resource production is to hydrolyze polysaccharides into fermentable sugar, and the degradation of lignocellulose requires the synergistic effect of cellulase and xylanase. However, xylanase was also reported as an ideal accessory enzyme for enhanced saccharification efficiency [37], which is due to the “xylanase boosting effect” [6] that breaks the crosslink between cellulose and xylan, and improves the accessibility of cellulase for better hydrolysis [6]. The hydrolysis of xylan was conducted with multiple xylanolytic enzymes [1], among that, the GH11 endo-xylanase was highly active on the backbone of xylan, but the efficient cleavage by endo-xylanase could also be impeded by the sub-chain group [10], which need the assistance of other xylanolytic enzymes. To establish a hyper-xylanase-producing platform, a series of efforts were conducted in *T. reesei* [22, 25], a potential fungus for the expression of multiple xylanolytic enzymes, to produce a highly active xylanase cocktail for xylan degradation.

In this study, first, the well-characterized transcription activator XYR1 and repressor ACE1 were manipulated. Beyond expectation, the combined strategy of upregulation of XYR1 and disruption of ACE1 resulted in slightly lower xylanase activity than that in C30OExyr1 (Fig. 2a), a transformant with XYR1 overexpression alone. ACE1 was identified as a repressor for cellulase production [20], and its deletion can increase the transcription level of cellulase and xylanase related gene. Moreover, a similar strategy conducted by Wang et al*.* [38] that combining XYR1 overexpression and ACE1 suppressing through RNAi resulted in higher xylanase and cellulase activities compared with sole XYR1 overexpression [38], which was opposite to our result. Besides, the expression of cellulase and xylanase was correlated with the abundance of XYR1 [31], and the expression of XYR1 in transformants could also be influenced by the locus of expression cassette and copy number. In our study, the copy number of *xyr1* was kept as the same in C30OExyr1 and C30OExyr1Δace1 (Fig. 1d), with the same insertion locus at the *ace1* site (Fig. 1a, b). While the transformant with overexpressed XYR1 cassette was randomly selected by Wang et al*.* [38], which was difficult to exclude the discrepancy in insertion locus. Moreover, the transcription level of *xyr1* in C30OExyr1Δace1 was much higher than that in C30OExyr1 at 24 h (Fig. 3c), the result was consistent with the previous study that ACE1 could antagonize *xyr1* transcription [30] through direct binding to the promoter of *xyr1* [39]. Besides, at 48 h, the transcription level of *xyr1* was comparative in two transformants. Surprisedly, the higher or comparative transcription of *xyr1* did not give a higher transcription of the xylanolytic gene in C30OExyr1Δace1 (Fig. 3b). ACE1 was reported that participate in the regulation through forming a protein complex with XYR1 at GGCTAA motif [29]. Indeed, the transcription regulation through regulator was more likely to form a polymer. In *Saccharomyces cerevisiae*, the Zn(II)_2_Cys_6_ type transcription regulator GAL4 was more likely to act as a dimer [40]. In *T. reesei*, the inverse repeat of GGC(A/T)_3_ was crucial for transcription of *xyn3* [41] and *xyn1* [29], suggesting that XYR1 could also functionally bind to the promoter as a homodimer. Meanwhile, the acidic activation domain of XYR1 was also verified for direct binding of ACE3 [42]. Thus, we speculated that in this study, the constitutive expression of XYR1 might disturb the distribution between XYR1 and ACE1, and further deletion of ACE1 could hinder the interaction between XYR1 and ACE1 which would lead to decreased production. In addition, the discrepancy between the two results of Wang et al*.* [38] and our study might be explained by that the suppression of *ace1* through RNAi only attenuates ACE1 function, but not to eliminate it. Our results give a new perspective that ACE1 might also be involved in the functional regulation of XYR1, but the mechanism still needs to be further investigated.

The transcription analysis of xylanolytic genes shows upregulation in C30OExyr1 (Fig. 3b), and the significantly upregulated genes were well correlated with the distribution of XYR1-binding sites except for *xyn2* (Fig. 3a), the gene encoding for major GH11 endo-xylanase in *T. reesei*. Mach-Aigner et al*.* also reported a less pronounced increase in the transcription level of *xyn2* compared to *xyn1* [30], which was in agreement with our data. Besides, partially constitutive expression of *xyn2* and other factors might also be involved in the direct regulation of *xyn2* other than XYR1 [21, 43]. Then, a strategy for enhanced endo-xylanase activity was performed by overexpression XYNII with the strongest *Pcbh1* promoter. Evidently, the *cbh1* locus was suitable for recombinant XYNII expression compared to the transformant with a randomly inserted cassette (Fig. 4b). Qin et al*.* show that the *cbh1* locus was highly transcribed [33], which was preferable for recombinant protein expression. In addition, the transcription level of *xyn2* was higher in C30OExyr1/xyn2Δcbh1 than that in C30OExyr1/xyn2 (Additional file 1: Figure S2. B), suggesting the higher xylanase production in C30OExyr1/xyn2Δcbh1 was partially due to the higher expression of XYNII in *cbh1* locus. Besides, the cassette inserted into *cbh1* locus with homologous recombination also resulted in the absence of major cellulase CBHI. CBHI was the most abundant cellulase under cellulose induction, which account for more than 50% of total secreted protein [44]. Theoretically, the absence of CBHI would leave more room for recombinant protein secretion, and release the ER stress for secreted protein [45]. In our study, improved xylanase production was also observed in CBHI deleted transformant (Fig. 4b) might due to the alleviation of feedback mechanism called RESS (REpression under Secretion Stress), which downregulates the transcription of cellulase and hemicellulase when suffered with ER stress [46]. In C30OExyr1/xyn2, decreased transcription of *xyn1* and *bxl1* was detected compared to that of C30OExyr1/xyn2Δcbh1 (Additional file 1: Figure S2. C), which probably result from the ER stress caused by recombinant XYNII expression, while in C30OExyr1/xyn2Δcbh1, ER stress was alleviated due to the absence of CBHI, which weaken the negative effect of RESS. We also used a CBHI deleted mutant C30Δcbh1 to evaluate the feedback caused by released ER stress, the result also showed the increased β-xylosidase activity compared to that of the parent strain (Additional file 1: Figure S3). Moreover, ER stress could also trigger the activation of UPR (Unfolded Protein Response), while the detection of the transcription of UPR related gene shows a higher transcription level in C30OExyr1/xyn2Δcbh1 than that in C30OExyr1/xyn2 (Additional file 1: Figure S2. A), which might be explained by the independent regulation pathway of UPR and RESS [47].

Meanwhile, the xylanase production in C30OExyr1/xyn2Δcbh1 was also applied using the economical and soluble carbon source lactose or glucose as the sole carbon source, and the xylanase production using lactose or glucose as carbon source was higher than that using xylan as an inducer (Fig. 6a). Li et al*.* [28] reported a relatively high XYNII activity of 9266 U/mL by constitutive overexpression of XYNII in *T. reesei*. In this study, we also overexpressed XYNII in *T. reesei* using *pdc* promoter (C30/pdcxyn2) according to Li et al*.* [28]. The xylanase production of C30/pdcxyn2 (1398 U/mL) was less than that of C30OExyr1/xyn2Δcbh1 (2310 U/mL) with glucose as carbon source. Moreover, the xylanase production in C30/pdcxyn2 was much lower than that achieved by Li et al*.* [28]. The fermentation medium used by Li et al*.* [28] was nutrient-rich which might result in higher xylanase output. Besides, the difference between transformants due to the insertion locus and copy number, and the substrate used for xylanase activity determined could also affect the final result. Obviously, in the same condition, C30OExyr1/xyn2Δcbh1 was preferable for xylanase production than C30/pdcxyn2 even in glucose medium. In addition, the lower xylanase production using lactose and glucose compared to that of Avicel could be conquered by fed-batch fermentation in a bioreactor, showing potential for applying on the industrial scale.

Afterward, the xylanase preparation with higher xylanase production achieved in C30OExyr1/xyn2Δcbh1 was tested for its saccharification ability with cellulase. In our study, a ratio of xylanase activity vs FPase activity (*X*/*F*) was set as a parameter to estimate the proper proportion of xylanase and cellulase under the same enzyme load. With higher proportion of xylanase, the xylan was hydrolyzed rapidly at early stage, improving accessibility for cellulase. However, with the same enzyme loaded, the proportion of cellulase and xylanase was critical for better saccharification. As shown in the result, the highest glucose yield was achieved at *X*/*F* = 500 under the same enzyme load (Fig. 7c). Besides, it was interesting that using a small amount of cellulase to improve saccharification with the higher xylanase proportion at *X*/*F* = 2000 (Fig. 7c), the efficient hydrolysis of xylan (Fig. 7b) loosen the complex structure of lignocellulose, and then, less amounts of cellulase could easily bind to the cellulose structure and make saccharification more efficient than that using cellulase alone, as indicated by Hu et al*.* [6]. Besides, the higher β-xylosidase activity in saccharification could attenuate inhibition of cellulase and xylanase activity by the decreased accumulation of xylooligosaccharides [48], showing potential for biomass conversion. Therefore, our study also provides a new strategy to keep the appropriate amount of xylanase cocktail to optimize the enzyme load for efficient lignocellulose saccharification. In this study, a significant increase in glucose yield was present with the increasing proportion of xylanase, which was partially attributed to the higher hemicellulose content (23.4%) in the pretreated lignocellulose. Liu et al*.* also reported increased saccharification of hemicellulose-rich corn straw by increase the abundance of XYNII through deletion of regulator SxlR [22], and Hu et al*.* also certified the decreased improvement of saccharification efficiency with the reduction of xylan content in different substrates [6]. However, due to different substrate characteristic and the different pretreated strategy, the hemicellulose content of different biomass was varied, so it was difficult to say whether the best ratio of *X*/*F* = 500 was also suitable for other biomass saccharification, but our work present here could also provide an effective strategy for higher xylanase production, which shows excellent performance in the saccharification of hemicellulose-rich lignocellulose. Besides, the comparison of saccharification results in different studies using xylanase addition also shows that our result gives a higher improvement in saccharification efficiency with the same enzyme load [6, 49]. Although higher improvement has also been achieved [36, 50, 51], further addition of xylanase with the same cellulase load increases costs (Table 1).

## Conclusion

A xylanase hyper-producing platform in *T. reesei* was established by constitutive expression of XYR1 and homologous expression of the native XYNII under the strong *cbh1* promoter. C30OExyr1/xyn2Δcbh1 exhibited improved xylanase-producing ability using several carbon sources, 5256 U/mL xylanase activity and 9.25 U/mL *p*NPXase activity were achieved on Avicel, associated with increased expression of several xylanolytic enzymes for efficient hydrolysis of xylan. Unexpectedly, deletion of *ace1* in C30OExyr1 did not give any improvement, which might be the result of the disturbed function of the complex formed between ACE1 and XYR1. Besides, a considerable amount of xylanase production was achieved using economical and soluble carbon sources, showing great potential for industrial fermentation. In addition, a 34.63% increase of APCS saccharification efficiency was attained using xylanase produced by C30OExyr1/xyn2Δcbh1 as accessory enzymes with *X*/*F* = 500. The novel platform with outstanding ability for crude xylanase cocktail production would greatly fit in biomass degradation and give a new perspective of further engineering in *T. reesei* for industrial purposes.

## Methods

### Strains and culture conditions

*Escherichia coli* TOP10, used as a cloning host for plasmid construction, was cultured at 37 ºC in Luria–Bertani (LB) medium. *Agrobacterium tumefaciens* AGL1 was used as a media for the transformation of *T. reesei* and was cultured in LB medium or inducing medium (IM) at 28 ºC according to purposes. *T. reesei* RUT-C30 (CICC 13052) was a parent strain that was kindly provided by Li [34]. *T. reesei* C30Δura3 was a uracil-deficient phenotype of RUT-C30 which was constructed previously in our lab. All the *T. reesei* were cultured on potato dextrose agar (PDA) plate at 28 ºC for conidiation. 10 mM uracil was added when uracil-deficient *T. reesei* Δura3 was cultured. For enzyme induction, 0.5 mL conidia suspension (10^7^/mL) was inoculated into 20 mL Sabouraud Dextrose Broth (SDB) and incubated for 40 h at 28 ºC with 200 rpm. After the accumulation of mycelia, the culture mixture was transferred into a 250 mL flask containing 50 mL of inducing medium with the inoculum rate of 10% (v/v). The flask was routinely cultured at 28 ºC with a constant shake of 200 rpm for 5 days. The Avicel-inducing medium was prepared as described previously [22], and wheat bran was added for sufficient nutrients. The glucose or lactose medium was similar to the Avicel-inducing medium that Avicel and wheat bran were replaced by lactose and glucose, respectively.

### Plasmid construction and fungal transformation

All the recombinant plasmid was constructed based on pCAMBIA1301G, which was derived from pCAMBIA1301 by replacing the promoter of hygromycin-resistant gene from *CaMV 35S* to a glyceraldehyde-3-phosphate dehydrogenase (*gpd*) promoter of *Aspergillus nidulans*.

For overexpression of *xyr1* (jgi|Trire2:122208) and deletion of *ace1* (jgi|Trire2:75418) in *T. reesei*, the *xyr1* coding sequence was amplified from genomic DNA of RUT-C30 with primers listed in Additional file 1: Table S2, and inserted into the *hyg* locus of pCAMBIA1301G resulting in a 3.8-kb *xyr1* overexpression cassette. Then, the 1.3-kb upstream and downstream of *ace1* was amplified from the genomic DNA of RUT-C30, combined with the *xyr1* overexpression cassette and *ura3* cassette, cloned to the backbone of pCAMBIA1301G using MultiF SeamLess Assembly Mix (ABclonal, Wuhan, China), resulting in pOExyr1Δace1. All the vectors constructed above were verified by PCR and sequencing.

For overexpressing major endo-xylanase gene *xyn2* (jgi|Trire2:123818) of *T. reesei* under *cbh1* promoter, 0.7-kb *xyn2* coding sequence including signal peptide sequence was amplified from cDNA of RUT-C30 with primers xyn2F/R (Additional file 1: Table S2). The 1.3-kb promoter and 1.1-kb terminal sequence of *cbh1* were amplified from genomic DNA, and the resulting three sequences were ligated through overlap extension PCR using the primers Pcbh1F/Tcbh1R. The *xyn2* expression cassette was ligated to the *Kpn*I and *SnaB*I sites of linearized pCAMBIA1301G by T4 ligase (NEB, England), resulting in pCbhxyn2 (Fig. 4a). pPdcxyn2 was constructed by changing the *cbh1* promoter with *pdc* promoter. To insert the expression cassette into *cbh1* locus, about 1.3-kb upstream of the *cbh1* promoter was cloned into pCbhxyn2, resulting in pCbhxyn2∆cbh1 (Fig. 4a).

The transformation of *T. reesei* was conducted by *Agrobacterium tumefaciens*-mediated transformation (ATMT) as described previously [52]. The selection of transformants was using MM plate [53] for uracil-deficient C30∆ura3, and PDA containing 20 mg/L hygromycin for others. The putative transformants and correct insert of the cassette were verified by PCR, single spore was isolated through successively streaking. The recombinant strains mentioned in this paper were listed in Additional file 1: Table S1.

### Biochemical analysis

Specifically, 1 mL mixture of shake flask fermentation was sampled every 24 h, the sample was centrifuged at 4 ºC, 12,000 rpm for 10 min to remove hypha pellet, and the supernatant was transferred to another tube and filtered for further analysis. The protein concentration was determined by Bradford Protein Assay Kit (Beyotime Biotechnology, Shanghai, China) as indicated by manual instruction. Cellulase and xylanase activity was determined by DNS method [16, 54] with a few modifications. In brief, 6.6 mg filter paper (Whatman No.1) was added into 180 μL 50 mM sodium citrate buffer with pH of 4.8, then 20 μL enzyme solution diluted to appropriate concentration was added to the mixture and incubated at 50 ºC for 60 min. The reaction was stopped by the addition of 300 μL alkaline 3,5-dinitrosalicylic (DNS) and boiled for 5 min, followed by immediate ice incubation. Then, the mixture was diluted fourfold and the absorbance at 540 nm was detected for correction of FPase activities. For xylanase activity determination, 180 μL 1% oat spelt xylan (TCL, Japan) in 50 mM sodium citrate buffer with pH of 5.3 was mixed with 20 μL diluted enzyme and incubated for 5 min, the following step was similar with cellulase activity analysis. One unit of enzyme activity was defined as the amount of enzyme that releases 1 μmol reducing sugar per minute. For β-xylosidase and β-glucosidase activity assay, 90 μL 4 mM *p*NPX or *p*NPG in 50 mM sodium acetate (pH 5.0) was mixed with 20 μL appropriately diluted enzyme and incubated for 10 min at 50 ºC. The reaction was stopped by adding 110 μL 2% sodium carbonate, and then, absorbance at 410 nm was detected. One unit of enzyme activity was defined as the amount of enzyme that releases 1 μmol *p*-nitrophenol per minute. SDS-PAGE analysis was performed on 9% Tris–HCl polyacrylamide gels using 3.75 μL supernatant of different *T. reesei* strains. All experiments were performed in three biological replicates.

### RNA extraction and RT-qPCR analysis

Culture mixtures were sampled at different time, mycelia were collected by centrifuged at 4 ºC, 12,000 rpm for 10 min and washed twice with distilled water, then the pellet was rapidly frozen in liquid nitrogen and stored at − 80 ºC for further analysis. RNA extraction was conducted using RNAiso plus (Takara, Japan) according to the manual’s instruction. The quality and quantity of extracted RNA were assessed by the absorbance at 260 nm on NanoDrop 8000 (Thermo Scientific, U.S.A.). Reverse transcription was performed using PrimeScript™ RT reagent Kit with gDNA Eraser (Perfect Real Time) (Takara, Japan) with total RNA extracted from different transformants cultured in inducing medium. Quantitative real-time PCR (RT-qPCR) was conducted in 20 μL per tube with 2*SG Fast qPCR Master Mix (High Rox) (Sangon Biotech, Shanghai, China) using primers listed in Additional file 1: Table S3, and the transcription level of *sar1* was used as inner standard. The PCR protocol run in the ABI StepOne instrument Plus (ABI, Germany) with software Version 2.3 (ABI, Germany) consisted of 3 min of initial denaturation at 95 °C, followed by 40 cycles of 3 s at 95 °C and 30 s at 60 °C. A melting curve was performed after each run to check the PCR product specificity. The data were calculated using (2^−ΔΔCT^) for relative quantification. All PCRs were carried out in triplicate on a plate.

### Copy number determination

For copy number determination, the genomic DNA of strain was extracted and applied for qPCR with the previous method [55], the gene *sar1* was represented as a single copy. The primer for copy number assay was also listed in Additional file: Table S3, with the qPCR procedure described above. Three biological replicates were performed for each reaction.

### In silico analysis of upstream of xylanolytic genes

To give a comprehensive view of the regulation of the xylanolytic gene, the 5′ upstream region was searched for each gene using the *T. reesei* genome database v2.0 (https://genome.jgi.doe.gov/Trire2/Trire2.home.html). The 1000 bp upstream from ATG of each gene was used for further analysis. The TATA box was identified by searching the TATAA motif about 100 bp upstream of ATG (the TATA box for *xyn3* was identified as TATA). For binding site exploration, the GGC(A/T)_3_ was searched upstream of the TATA box, the motif in two strands were both under consideration. For putative ACE1 binding site searching, the core element of AGGCA was used for identifying corresponded ACE1-binding sites.

### Saccharification of alkali pretreated corn stover

The corn stover was purchased from the country of Jiangsu (China), then, treated with 2% NaOH with a load of 10% (w/v) at 121 ºC for 30 min. The mixture was washed with distilled water until neutral pH, and dried at 60 ºC for 24 h, then smashed for better utilization. The alkali pretreated corn stover (APCS) was stored at 4 ºC for further study. The content of APCS was determined using the NREL method [56], which contains 57.2% cellulose and 23.4% hemicellulose. The total activity of cellulase and xylanase was calculated through the formula below, and the total xylanase activity vs total cellulase activity was set as *X*/*F* to evaluate the amount of cellulase and xylanase in each reaction. The lowest *X*/*F* of 24 and highest of 2770 represented the sole reaction by cellulase and xylanase preparation, respectively. In addition, a series ratio from 24 to 2770 was set to systematically determine the best ratio for saccharification.

$${\text{X}}/{\text{F}}={\frac{{\text{Xylanase activity}}_{\text{cellulase preparation}}+{\text{Xylanase activity}}_{\text{xylanase preparation}}}{{\text{FPU}}_{\text{cellulase preparation}}+{\text{FPU}}_{\text{xylanase preparation}}}}$$

The commercial cellulase used in this study was kindly provided by Sunson (NingXia, China), and the xylanase cocktail was prepared by culturing C30OExyr1/xyn2Δcbh1 in Avicel medium for 5 days. For saccharification experiments, 500 ± 0.5 mg APCS was mixed with 4 mg (protein concentration) enzyme preparation (Table 2) in a 25 mL flask and finally making up to 10 mL with 50 mM sodium acetate (pH 5.0). Besides, the β-glucosidase (Sunson) was added at CBU/FPU = 2 to limit the end-production inhibition. The reaction was performed at 50 ºC, 150 rpm for 72 h. Samples were collected at 2, 6, 12, 24 and 72 h, and the sugar content was analyzed by High-Performance Liquid Chromatography (HPLC) with a Bio-Rad Aminex HPX-87H column and Agilent 1260 Infinity II refractive index detector. 5 mM H_2_SO_4_ was used as mobile phase at a flow rate of 0.5 mL/min. All the experiments were conducted in three biological replicates.

## Supplementary Information

**Additional file 1: Table S1.** Strains used in this study. **Table S2.** Primers used for recombinant plasmid construction. **Table S3.** Primers used for RT-qPCR and copy number determination. **Table S4.** Comparison of xylanase production in different organisms. **Table S5.** The hydrolysis efficiency of lignocellulose. **Figure S1.** The transcription level of xylanolytic genes of C30OExyr1/xyn2Δcbh1 in lactose and glucose medium. **Figure S2.** The transcription level of relative genes in C30OExyr1/xyn2 and C30OExyr1/xyn2Δcbh1. **Figure S3.** The β-xylosidase (*p*NPXase) activity of the C30Δcbh1 and the parent strain.

## Data Availability

All data generated or analyzed during this study are included in this published article and its Additional file 1.

## Abbreviations

APCS: Alkali pretreated corn stover
ATMT: *Agrobacterium tumefaciens*-Mediated transformation
CCR: Carbon catabolite repression
HPLC: High-Performance Liquid Chromatography
MM: Minimal medium
RESS: REpression under Secretion Stress
SDB: Sabouraud Dextrose Broth
UPR: Unfolded Protein Response
XBD: XYR1-binding sites
