# Supplementary material for: Rational engineering of xylanase hyper-producing system in Trichoderma reesei for efficient biomass degradation
Source: Biotechnol Biofuels. 2021 Apr 8;14:90. doi: 10.1186/s13068-021-01943-9 (PMC8033665; doi:10.1186/s13068-021-01943-9)
Supplement: Supplementary file 1 — Additional file 1: Table S1. Strains used in this study. Table S2. Primers used for recombinant plasmid construction. Table S3. Primers used for RT-qPCR and copy number determination. Table S4. Comparison of xylanase production in different organisms. Table S5. The hydrolysis efficiency of lignocellulose. Figure S1. The transcription level of xylanolytic genes of C30OExyr1/xyn2Δcbh1 in lactose and glucose medium. Figure S2. The transcription level of relative genes in C30OExyr1/xyn2 and C30OExyr1/xyn2Δcbh1. Figure S3. The β-xylosidase (pNPXase) activity of the C30Δcbh1 and the parent strain. [file 13068_2021_1943_MOESM1_ESM.docx]

**Additional file 1**

**Rational engineering of xylanase hyper-producing system in *Trichoderma reesei* for efficient biomass degradation**

Su Yan, Yan Xu, Xiao-Wei Yu^*^

*Key Laboratory of Industrial Biotechnology, Ministry of Education, School of Biotechnology, Jiangnan University, Wuxi 214122, P.R. China*

^*^ Corresponding author

E-mail addresses: yuxw@jiangnan.edu.cn (X.W. Yu)

**Table S1** Strains used in this study

| **Strains** | **Genotype** | **Source** |
| --- | --- | --- |
| TOP10 | *Escherichia coli* used for Cloning | Stored in lab |
| AGL1 | *Agrobacterium tumefaciens* AGL1 typically used for ATMT | Stored in lab |
| RUT-C30 | *Trichoderma reesei* CICC 13052 | See in paper |
| C30Δura3 | *ura3*^-^ | Stored in lab |
| C30/xyn2 | *ura3*^+^ *hyg*^+^ *Pcbh1-xyn2-Tcbh1* | This study |
| C30/xyn2Δcbh1 | *ura3*^+^ *hyg*^+^ Δ*cbh1*::*Pcbh1-xyn2-Tcbh1* | This study |
| C30/pdcxyn2 | *ura3*^+^ *hyg*^+^ *Ppdc-xyn2-Tcbh1* | This study |
| C30OExyr1 | *ura3*^+^ *Pgpd-xyr1* | This study |
| C30OExyr1Δace1 | *ura3*^+^ Δ*ace1*::*Pgpd-xyr1* | This study |
| C30OExyr1/xyn2 | *ura3*^+^ *hyg*^+^ *Pgpd-xyr1*; *Pcbh1-xyn2-Tcbh1* | This study |
| C30OExyr1Δace1/xyn2 | *ura3*^+^ *hyg*^+^ Δ*ace1*::*Pgpd-xyr1*; *Pcbh1-xyn2-Tcbh1* | This study |
| C30OExyr1/xyn2Δcbh1 | *ura3*^+^ *hyg*^+^ *Pgpd-xyr1*; Δ*cbh1*::*Pcbh1-xyn2-Tcbh1* | This study |
| C30OExyr1Δace1/xyn2Δcbh1 | *ura3*^+^ *hyg*^+^ Δ*ace1*::*Pgpd-xyr1*; Δ*cbh1*::*Pcbh1-xyn2-Tcbh1* | This study |

**Table S2** Primers used for recombinant plasmid construction

| **Primers** | **Sequence (5’-3’)** | **Target** |
| --- | --- | --- |
| *Pcbh1* F | TCGGTACCCATGAAAGGCTATGAGAAATTCTGGAGACG (*K*pn Ⅰ) | *cbh1* promoter |
| *Pcbh1* R | **AAGGAGACCATGATGCGCAGTC**CGCGGTT |  |
| *Xyn2* F | **GACTGCGCATCATGGTCTCCTT**CACCTCCCTCC | *xyn2* coding sequences |
| *Xyn2* R | **AGCTGAGCTCTTAGC**TGACGGTGATGGAAGC |  |
| *Tcbh1* F | **AGCTAAGAGCTCAGCT**CCGTGGCGAAAG | *cbh1* terminator (right arm) |
| *Tcbh1* R | AATACGTAAACTCGTGCTCTCTCGCG (*S*naB Ⅰ) |  |
| *Ppdc* F | AATTGGTACCAGGACTTCCAGGGCTACTTGGC (*K*pn Ⅰ) | *pdc* promoter |
| *Ppdc* R | **GGGAGGTGAAGGAGACCAT**GATTGTGCTGTAGCTGCGCTG |  |
| *Cbh1L* F | **TAATTCGGGGGAATTCGCGTA**CATCCATCATCACGCACGAC | *cbh1* left arm |
| *Cbh1L* R | **AAATCCAGATCGAGCTCTACG**AACAAACAAGCGACCCAATTGG |  |
| *Poly* F | **CGTAGAGCTCGATCTGGATTT**TAGTACTGGATTTTGGTTTTAG | Backbone of pCAMBIA1301G |
| *Lb* R | **ACGCGAATTCCCCCGAATTA**ATTCGGCGTTAATTCAGT |  |
| *Xyr1* F | **CGCTTGAGCAGACATCACC**ATGTTGTCCAATCCTCTCCGTCG | *xyr1* coding sequences |
| *Xyr1* R | **TCCGGTCGGCATCTACT**TTAGAGGGCCAGACCGGTTCC |  |
| *Ura3* F | **TAACGGTGAGACTAGCGGCC**G | *ura3* complementary cassette |
| *Ura3* R | **GTTTCCAGGTGCTCCTGGG**CC |  |
| *Ace1L* F | **ATTCGGGGGAATTCGCGT**GCTTGCACTGCTGATTTCCGAC | *ace1* left arm |
| *Ace1L* R | **ATGAAGCTCCATATTCTCCG**CAAGCCCGAGATAGACGAAGGC |  |
| *Ace1R* F | **CCCAGGAGCACCTGGAAAC**AGATGCTGCAAACGCTTATCCAC | *ace1* right arm |
| *Ace1R* R | **GGATTCAATCTTAATACGTACCTG**CACAGAGGTACGCAGCAAAGAG |  |
| *P1* | CTGCGAACATGGCCTGTCTCT | PCR verification of *ace1* |
| *P2* | AAAAGGGAAGCCTTGCCGAC |  |
| *P3* | GGGGTTAACATGGACGAGAACAG | PCR verification of left arm |
| *P4* | CTCTCTCGGACATATTCGCA |  |
| *P5* | TAACCATGGCACCACACCCGACGCTCAA | PCR verification of right arm |
| *P6* | AATATCGTCGGACATATCGTGGGC |  |

* Sequence with underline means restriction sites, and the reverse complementary sequences were shown in bold font. F means forward primer and R means reverse primer.

**Table S3.** Primers used for RT-qPCR and copy number determination.

| **Primer** | **Forward (5’-3’)** | **Reverse (5’-3’)** |
| --- | --- | --- |
| *xyr1* | TACCAAGTGCGATGGCTTAC | CTCTCTCGGACATATTCGCA |
| *ace1* | AAGACCCTGATCTTCATGGC | ATTCGACTGTCGCTTGAATG |
| *xyn1* | GGTTGGACGACTGGATCT | GGTTGTCCTCCATGATGTAG |
| *xyn2* | CGAGTACTACATCGTCGAGAA | CGTGCGGTAAATGTCGTAG |
| *xyn3* | GTACAAGGGCAAGATTCGT | AAGGCAATCGAGACAAACT |
| *xyn4* | GGGTGGCGAGAGATACTT | TGCCCACTCAGTCTGTATG |
| *bxl1* | ACATCAAGCCTGGTCACTC | CAGACTCGTCGGTGTTCA |
| *axe1* | TCTTCGATAATGCCCTCTGC | TTATAGGGCAGGCCATGAATG |
| *abf1* | ATGGAGTTTTCGTGTCTCCA | TCTCGGCGTTGCCATAGT |
| *sar1* | TGGATCGTCAACTGGTTCTACGA | TGTGTAGCAACGTGGTCTTT |
| *hac1* | CCTGCAGTGTCAATCGGT | AATGGCCGATGCTGAAAG |
| *bip1* | GATGCCAACGGTATCCTCA | TGCGGTCAATCTCCTCCT |
| *pdi1* | GTTGTCGTTGCCCACTCTTAC | AGTCGCTCTTGGCATACAGG |
| g*xyr1^*^* | CGAAGCGTCCTTCTTGACGA | GTGGATCAGGGGCTGAAGAA |
| g*sar1*^*^ | GCCGACTCTCCACCCTAGTA | GAAACCCTCCACAAACCCCA |

Asterisk means the primer used for copy number determination.

**Table S4.** Comparison of xylanase production in different organisms

| **Carbon source** | **Expression host** | **Fermentation method** | **Xylanase activity (U/mL)** | **β-xylosidase activity (U/mL)** | **Reference** |
| --- | --- | --- | --- | --- | --- |
| 3% Avicel | *Trichoderma reesei* | Flask | 5256 | 9.25 | This study |
| 3.6% Cellulose | *Penicillium oxalicum* | Flask | ~950 | 15.05 | [1] |
| 2% Avicel | *Trichoderma orientalis* | Flask | ~18 | ~1 | [2] |
| 7% Glucose | *Trichoderma reesei* | Flask | 9266 | - | [3] |
| 5% Glucose | *Pichia pastoris* | Bioreactor | 4115.2 | - | [4] |
| Methanol | *Pichia pastoris* | Bioreactor | 1650 | - | [5] |
| 1% Beech xylan | *Trichoderma reesei* | Bioreactor | 1373 | - | [6] |

1. Ye Y, Li X, Cao Y, Du J, Chen S, Zhao J. A beta-xylosidase hyper-production *Penicillium oxalicum* mutant enhanced ethanol production from alkali-pretreated corn stover. Bioresour Technol. 2017;245:734-742.

2. Xue Y, Han J, Li YY, Liu J, Gan LH, Long MN. Promoting cellulase and hemicellulase production from *Trichoderma orientalis* EU7-22 by overexpression of transcription factors Xyr1 and Ace3. Bioresour Technol. 2020;296:9.

3. Li J, Wang J, Wang S, Xing M, Yu S, Liu G. Achieving efficient protein expression in *Trichoderma reesei* by using strong constitutive promoters. Microb Cell Fact. 2012;11:84.

4. Wang J, Liu YJ, Yang YZ, Bao CL, Cao YH. High level expression of an acidic thermostable xylanase in *Pichia pastoris* and its application in weaned piglets. J Anim Sci. 2019; 98:1.

5. Long LF, Zhang YB, Ren HY, Sun HY, Sun FBF, Qin WS. Recombinant expression of *Aspergillus niger* GH10 endo-xylanase in *Pichia pastoris* by constructing a double-plasmid co-expression system. J Chem Technol Biotechnol. 2020;95:535-543.

6. Bailey MJ, Buchert J, Viikari L. Effect of Ph on Production of Xylanase by *Trichoderma-Reesei* on Xylan-Based and Cellulose-Based Media. Appl Microbiol Biotechnol. 1993;40:224-229.

**Table S5.** The hydrolysis efficiency of lignocellulose

| **X/F^a^** | **24** | **50** | **100** | **200** | **500** | **1000** | **1500** | **2000** | **2770** |
| --- | --- | --- | --- | --- | --- | --- | --- | --- | --- |
| **Glucose (mg/mL)** | 13.52 | 14.98 | 15.54 | 17.05 | 18.20 | 17.65 | 16.34 | 15.02 | 11.55 |
| **Cellulose hydrolysis^b^** | 47.23% | 52.34% | 54.31% | 59.58% | 63.59% | 61.68% | 57.10% | 52.48% | 40.37% |
| **Xylose (mg/mL)** | 4.51 | 5.95 | 6.22 | 7.35 | 8.48 | 9.06 | 9.23 | 9.34 | 9.48 |
| **Xylan hydrolysis^c^** | 43.35% | 57.17% | 59.73% | 70.63% | 81.49% | 87.08% | 88.67% | 89.71% | 91.08% |

**^a^** X/F means the xylanase activity vs FPase activity in the saccharification system. And equal amount of enzyme (8 mg/g APCS) was loaded for each reaction.

**^b^** Cellulose hydrolysis was calculated using (glucose content**/**cellulose content in biomass) * 100%

**^c^** Xylan hydrolysis was calculated using (xylose content**/**xylan content in biomass) * 100%

**Figure S1.**


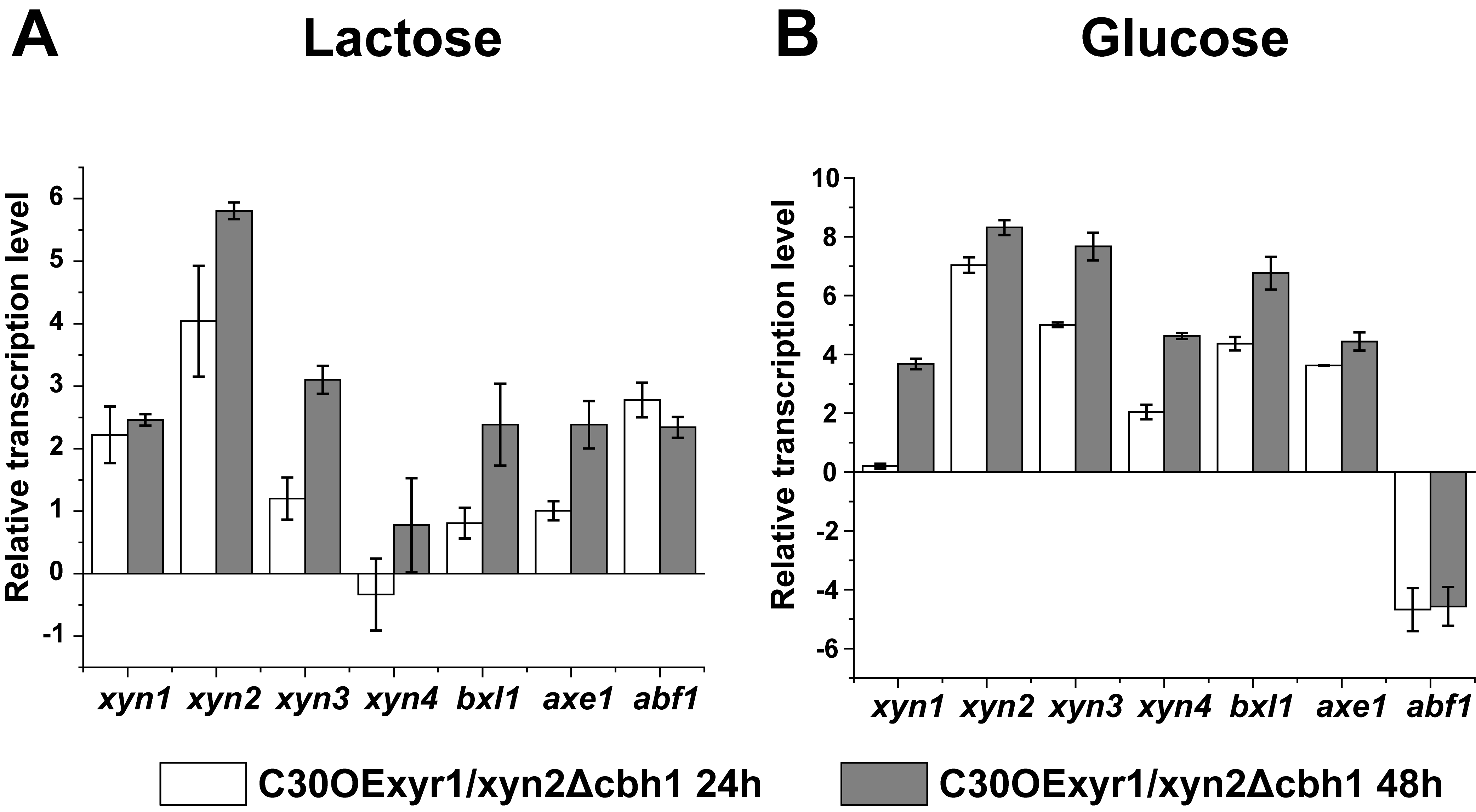


**Figure S1.** The transcription level of xylanolytic genes of C30OExyr1/xyn2Δcbh1 in lactose and glucose medium. The strain C30OExyr1/xyn2Δcbh1 was cultured with SDB for 40 h and transferred to the medium using 5% lactose (**A**) or 5% glucose (**B**) as sole carbon source, respectively. The sample at 24 h and 48 h were analyzed for RT-qPCR. The transcription level of the corresponding gene of RUT-C30 was set as 1, and the transcription level was indicated by log_2_(fold change).

**Figure S2.**


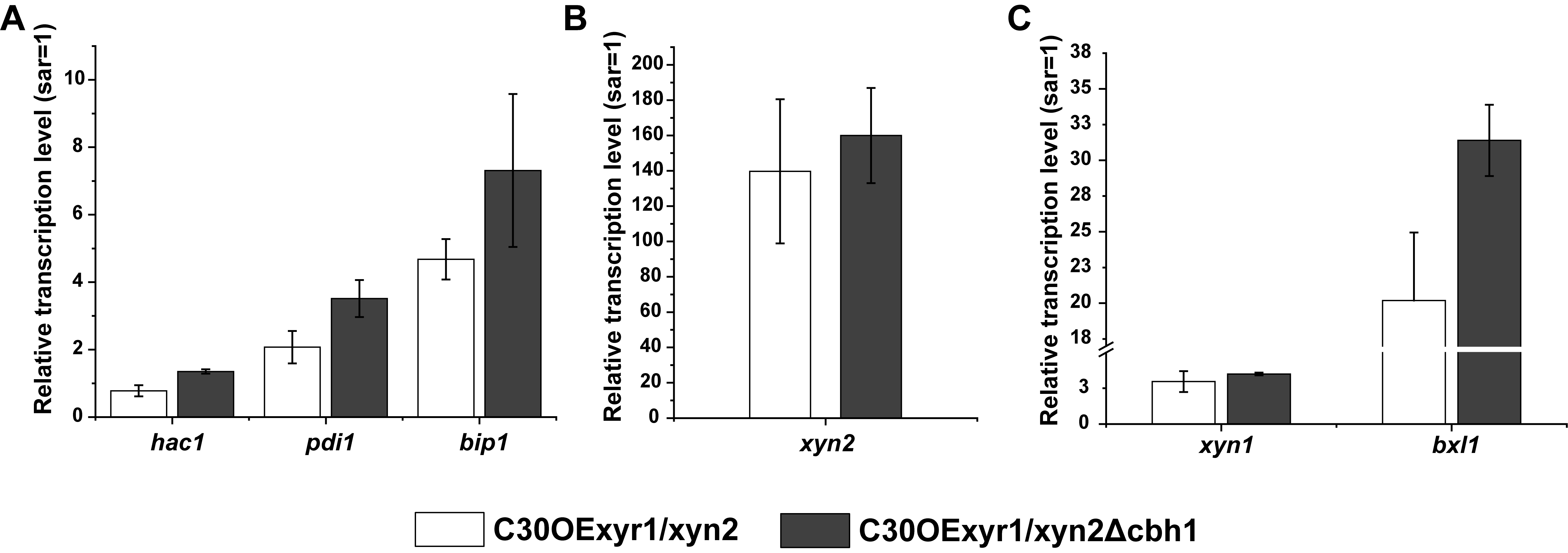


**Figure S2.** The transcription level of relative genes in C30OExyr1/xyn2 and C30OExyr1/xyn2Δcbh1. The two strains were both induced with Avicel, and samples at 24 h were analyzed for RT-qPCR. **A**, the relative transcription level of UPR related genes. **B**, the relative transcription level of *xyn2*. **C**, the relative transcription level of *xyn1* and *bxl1.* The transcription level was measured using the 2^^-ΔΔCT^ method, and the transcription level of *sar1* was used for normalization.

**Figure S3.**

**Figure S3.** The β-xylosidase (*p*NPXase) activity of the C30Δcbh1 and the parent strain. The C30Δcbh1 was constructed previously by insertion of URA3 marker into the *cbh1* locus of C30Δura3. The two strains were both cultured with Avicel and the supernatant after 5 day was assayed for β-xylosidase (*p*NPXase) activity.
